# Response to the comment on: Comparison of creatinine- and cystatin C–based definitions of acute kidney injury in neonates with congenital diaphragmatic hernia

**DOI:** 10.1007/s00467-026-07224-7

**Published:** 2026-02-19

**Authors:** Judith Leyens, Lukas Schroeder

**Affiliations:** 1 Department of Neonatology and Pediatric Intensive Care Medicine, University of Bonn, University Children’s Hospital of Bonn, Bonn, Germany; 2 Division of Neonatology, Department of Pediatrics, BC Women’s and Children’s Hospital, University of British Columbia, Vancouver, Canada; 3 Division of Congenital Malformations, Center for Rare Diseases, University of Bonn, University Hospital of Bonn, Bonn, Germany

Dear Editors,

We thank Khan et al. [1] for their thoughtful appraisal of our study [2] and for highlighting important methodological considerations.

First, although cystatin C (CysC) measurements were not available for the entire cohort, this primarily reflects a change in local protocol rather than selective testing of critically ill infants. Since early 2018, both serum creatinine (SCr) and CysC have been included in the standard order set for all neonates with congenital diaphragmatic hernia (CDH). In infants with milder disease, protocolized blood sampling was often discontinued earlier for clinical and economic reasons, resulting in fewer CysC follow-up measurements compared with SCr. Consequently, CyNA-defined AKI may have been underestimated rather than overestimated. Baseline illness severity was assessed using antenatal risk stratification and postnatal clinical variables, and multivariable analyses adjusted for key indicators of disease severity.

Second, the weekly recalculated baseline approach was chosen to account for dynamic neonatal kidney physiology and prolonged intensive care courses typical for CDH. As both SCr and CysC physiologically decline during the first weeks of life, relying solely on early postnatal values risks underdetection of later AKI episodes [3]. Applying the same methodology across biomarkers enables consistent comparative interpretation across AKI definitions.

Third, we describe associations rather than causal relationships between AKI severity and outcomes. Mortality analyses were therefore interpreted descriptively and in the context of disease severity. The contribution of SCr- versus CysC-defined AKI episodes to long-term outcomes, including kidney and cardiovascular morbidity, remains unknown. We totally agree with the authors [1] that prospective studies with protocolized biomarker sampling and longitudinal follow-up are required to validate these associations in the CDH population.

## Data Availability

The data that support the findings of this study are available on request from the corresponding author. The data are not publicly available due to privacy or ethical restrictions.

## References

[1] Khan MS, Tausif MT (2026) Comment on: Comparison of creatinine- and cystatin C– based definitions of acute kidney injury in neonates with congenital diaphragmatic hernia. Pediatr Nephrol. 10.1007/s00467-026-07196-8 41652246 10.1007/s00467-026-07196-8

[2] Leyens J, Gerschlauer J, Berg C, Strizek B, Mueller A, Kipfmueller F, Schroeder L (2026) Comparison of creatinine- and cystatin C-based definitions of acute kidney injury in neonates with congenital diaphragmatic hernia. Pediatr Nephrol. 10.1007/s00467-026-07156-2 41580574 10.1007/s00467-026-07156-2 PMC13197386

[3] Xu X, Nie S, Xu H, Liu B, Weng J, Chen C, Liu H, Yang Q, Li H, Kong Y, Li G, Wan Q, Zha Y, Hu Y, Xu G, Shi Y, Zhou Y, Su G, Tang Y, Li Y, Su L, Chen R, Cao Y, Gao P, Zhou S, Zhang X, Luo F, Xu R, Gao Q, Hou FF (2023) Detecting neonatal AKI by serum cystatin C. J Am Soc Nephrol 34:1253–1263. 10.1681/ASN.0000000000000125 36977125 10.1681/ASN.0000000000000125 PMC10356146

